# Aphid resistance in *Capsicum* maps to a locus containing LRR-RLK gene analogues

**DOI:** 10.1007/s00122-019-03453-7

**Published:** 2019-10-08

**Authors:** Mengjing Sun, Roeland E. Voorrips, Wendy van’t Westende, Martijn van Kaauwen, Richard G. F. Visser, Ben Vosman

**Affiliations:** Plant Breeding, Wageningen University and Research, P.O. Box 386, 6700 AJ Wageningen, The Netherlands

## Abstract

**Key message:**

A QTL for aphid resistance on pepper chromosome 2 was identified and validated. This QTL affects aphid survival and reproduction, and was fine mapped to a locus containing LRR-RLK analogues.

**Abstract:**

*Myzus persicae* is one of the most threatening insect pests that adversely affects pepper (*Capsicum*) cultivation. Resistance to aphids was previously identified in *Capsicum baccatum*. This study aimed at elucidating the genetics of aphid resistance in *C. baccatum*. A QTL analysis was carried out for *M. persicae* resistance in an F_2_ population derived from an intraspecific cross between a highly resistant plant and a susceptible plant. Survival and reproduction were used as resistance parameters. Interval mapping detected two QTLs affecting aphid survival (*Rmpas-1*) and reproduction (*Rmprp-1*), respectively, both localized in the same area and sharing the same top marker on chromosome 2. Use of this marker as co-factor in multiple-QTL mapping analysis revealed a second, minor QTL (*Rmprp-2*) only affecting aphid reproduction, on chromosome 4. Fine mapping confirmed the effects of *Rmpas-1* and *Rmprp-1* and narrowed the major QTL *Rmprp-1* down to a genomic region of 96 kb which is predicted to encode four analogues of resistance genes of the receptor-like kinase family containing a leucine-rich repeat domain (LRR-RLKs). This work provides not only initial information for breeding aphid-resistant pepper varieties, but also forms the basis for future molecular analysis of gene(s) involved in aphid resistance.

**Electronic supplementary material:**

The online version of this article (10.1007/s00122-019-03453-7) contains supplementary material, which is available to authorized users.

## Introduction

Pepper (*Capsicum* spp.) is one of the economically most important and widely cultivated vegetable crops. However, its cultivation is constrained by aphids (Frantz et al. 2004; Herman et al. 2008). Aphids can damage pepper plants in many ways, leading to chlorosis, defoliation, wilting and flower abortion. Aphids also can cause a reduction in the photosynthetic capacity and fruit quality through the stimulation of moulds that grow on the honeydew. But the most important damage to pepper plants is done indirectly by the viruses that are vectored by the aphids (Black et al. 1991; Kennedy et al. 1962; Kenyon et al. 2014).

Among the different aphid species feeding on pepper, the green peach aphid (GPA), *Myzus persicae*, is one of the most threatening (Fereres et al. 1993). The control of GPA population development is currently mainly carried out using insecticides. However, given the fact that more and more aphid species have developed resistance against the pesticides (Bass et al. 2014; Devonshire et al. 1998; Foster et al. 2000) and that insecticides negatively affect the environment, varieties resistant to the GPA may be a more promising alternative. Resistance to aphids not only reduces the size of the aphid population on pepper (Frantz et al. 2004; Sun et al. 2018), but may also decrease the percentage of plants infected with viruses (Radcliffe and Ragsdale 2002). In several plant species, quantitative trait loci (QTLs) for resistance against aphids have been detected. Aphids for which resistance QTLs have been found include the soybean aphid *Aphis glycines* (Kim et al. 2010), the pea aphid *Acyrthosiphon pisum* (Stewart et al. 2009), the cotton aphid *Aphis gossypii* (Boissot et al. 2010; Liang et al. 2016), the potato aphid *Macrosiphum euphorbiae* (Rossi et al. 1998) and the Russian wheat aphid *Diuraphis noxia* (Aykut Tonk et al. 2016). However, only a few QTLs for resistance to GPA were found, most of which were in peach (Lambert and Pascal 2011; Pascal et al. 2017; Sauge et al. 2012, 2004) and *Arabidopsis* (Kloth et al. 2017; Pfalz et al. 2009; Thoen et al. 2017). No aphid resistance QTL has been described in pepper so far.

Until now two genes affecting aphid performance have been cloned based on identified QTLs (Pauquet et al. 2004; Vos et al. 1998). Both of them, the tomato *Mi-1.2* gene and the melon *Vat* gene, conferring resistance to *M. euphorbiae* and *A. gossypii,* respectively, are of the NBS-LRR type, sharing motifs with many disease resistance genes (Belkhadir et al. 2004; Broekgaarden et al. 2011). This type of genes code for proteins that can recognize an effector from an external pathogen or pest and activate a specific immune response in the host plant according to the gene-for-gene principle, which is called effector-triggered immunity (ETI) (Jones and Dangl 2006; Van Der Biezen and Jones 1998). Next to ETI, the innate immune system of plants consists of another layer, the microbe-associated molecular pattern (MAMP)-triggered immunity (MTI) (Jones and Dangl 2006; Silva Couto and Zipfel 2016) or the herbivore-associated molecular pattern (HAMP)-triggered immunity in case of herbivores (Bonaventure et al. 2011). The MAMPs and HAMPs can generally be recognized by pattern recognition receptors (PRRs) in plants, which often belong to the family of receptor-like kinases (RLKs) (Jones and Dangl 2006; Zipfel 2014). Several resistance genes from the RLK family have been identified, which provide protection against pathogens (Fradin et al. 2009; Gómez-Gómez and Boller 2000; Krol et al. 2010), nematodes (Mendy et al. 2017) and insects (Liu et al. 2015). However, no PRR gene has been found that is involved in plant resistance against aphids. Only one RLK gene was reported to act as a co-receptor of other unknown PRRs and play a role in aphid resistance in *Arabidopsis* (Prince et al. 2014). Fine mapping resistance QTLs may help to identify more genes involved in aphid resistance and provide clues to whether they are NBS-LRRs or RLKs type of genes.

In a previous paper, we have described the screening and characterization of several sources of GPA resistance in *Capsicum* species (Sun et al. 2018). Three accessions of *Capsicum baccatum* were shown to be resistant or intermediately resistant to GPA by negatively affecting aphid survival and reproduction. Among them, accession PB2013071 showed the highest level of resistance, which severely impaired phloem uptake by the aphid and induced callose deposition in the sieve elements during aphid feeding. Our current study aimed at elucidating the genetics of GPA resistance in accession PB2013071 through a QTL mapping approach followed by fine mapping. This work will enable us to discover the gene underlying GPA resistance, which is useful information for breeding aphid-resistant pepper varieties.

## Materials and methods

### Plant materials, growing conditions and aphid population

The plant materials were obtained from the collection of Plant Breeding in Wageningen University and Research, Wageningen, NL. Aphid-resistant accession PB2013071 and susceptible accession PB2013046 of *C. baccatum* were described previously (Sun et al. 2018). An F_2_ mapping population of 192 plants was obtained after selfing a single F_1_ plant obtained from the cross between PB2013046 as female parent and PB2013071 as male parent. First-generation inbred lines of the resistant (PB2014009) and susceptible (PB2014005) parent were obtained by self-pollination. Eight F_3_ lines derived from four F_2_ plants that were homozygous for the resistance allele and four F_2_ plants that were homozygous for the susceptibility allele in the 2-LOD confidence interval of the major QTL were obtained by selfing and used for QTL validation. For fine mapping of the resistance gene, we used F_3_ line PB2016027 which was obtained after selfing an F_2_ plant that was heterozygous for the 2-LOD interval of the major QTL. In the F_3_ line, we selected 230 recombinants for fine mapping. Five plants from F_3_ line PB2016027 that were homozygous for the resistance allele and five plants that were homozygous for the susceptibility allele in the 2-LOD interval of the major QTL were also kept and used for evaluation and validation of the QTL.

For all experiments, seedlings were transplanted into 17-cm pots with potting compost 2 weeks after sowing and grown in a standard greenhouse at 19–21 °C, 60–70% RH and an L16:D8 photoperiod at Unifarm, Wageningen University and Research, Wageningen, NL. During growth and testing, plants were watered every other day without any pest control and fertilized with 2.5 mg l^−1^ Kristalon Blauw (pH = 5.5, N–P–K, 4–1–7; Hydro Agri, Rotterdam, Netherlands) every 2 weeks.

The GPA (*M. persicae*) population used was the same as the one we used previously (Sun et al. 2018). It was reared on *C. baccatum* accession PB2013046 and was maintained under the same conditions as the pepper plants.

### Resistance evaluation by clip cage tests

Resistance evaluations were carried out when the plants were 7 weeks old in the greenhouse of Unifarm, Wageningen University and Research, Wageningen, NL. Every plant under evaluation received three clip cages containing five 1-day-old nymphs. After 12 days, the living and dead aphids as well as the new nymphs produced in each clip cage were counted.

For the phenotyping of the F_2_ population, in October 2015 192 F_2_ plants were randomized and equally divided over two greenhouse compartments next to each other with the same climate conditions. For the major QTL validation, five plants of eight F_3_ lines either homozygous for the resistance or susceptibility allele over the 2-LOD QTL interval were randomized in one compartment in August 2016. For fine mapping of the major resistance gene, in July 2017 230 recombinants from F_3_ line PB2016027 together with ten homozygous plants (five with the resistance allele and five with the susceptibility allele over the 2-LOD QTL interval) from the same line were randomized and equally divided over the two compartments with the same climate conditions. Five plants of the two first-generation parental inbred lines, PB2014009 and PB2014005, were included as reference in every evaluation and randomized together with the other materials.

For statistical analysis, the observations from the three clip cages on a plant were always combined. Clip cages with less than four aphids (dead or alive) were discarded, as were data on plants with less than two remaining clip cages. Two resistance parameters, survival of the original nymphs and the number of next-generation nymphs produced per aphid, were analysed. Survival data were obtained by dividing the number of living aphids by the total number of aphids. The number of next-generation nymphs was divided by the average number of living aphids present, which was calculated as (2*living aphids + dead aphids)/2. Data were transformed as follows: survival as arcsin(sqrt(*x*)) and next-generation nymphs as sqrt(*x*). Transformed data were used in statistical analysis and QTL mapping. In the two experiments where the plants were divided over two compartments, no significant compartment effect was detected (*P* > 0.05 in both cases) and consequently the compartment effect was ignored in further statistical analyses and QTL mapping. Data from the QTL validation experiment with either the resistance or susceptibility allele were analysed using ANOVA combined with a LSD test (*P* < 0.05). For comparing the parental inbred lines in the QTL mapping and fine mapping experiments, a *T* test was used. The Pearson correlation was used to evaluate the correlation between aphid survival and reproduction.

### DNA extraction, molecular markers and genetic linkage map construction

Samples of newly expanded leaves of plants were collected and stored at – 80 °C until DNA extraction. Collected samples were ground using a TissueLyser II (Qiagen, USA). After being ground, genomic DNA was extracted using the CTAB method (Fulton et al. 1995). The DNA quantity and quality were determined by NanoDrop 1000 V.3.7 (Thermo, USA).

DNA from the F_1_ plant used for making the F_2_ population was sequenced by one lane Illumina HiSeq2500 (60 Gb, 2 × 125 nt Paired End). Reads were mapped to the reference genome *C. annuum* ‘CM334’ v.1.55 (Kim et al. 2014) (https://www.peppergenome.snu.ac.kr/) using BWAmem (Li 2013). Single nucleotide polymorphisms (SNPs) were identified using Freebayes (Garrison and Marth 2012). A total of 167 evenly spaced SNP markers (Table S1) were selected and named based on their physical position on the *C. annuum* genome sequence ‘CM334’ v.1.55 (Kim et al. 2014).

DNA solutions of F_2_ plants were prepared for genotyping through the KASP™ technology (KBioscience, UK), which was carried out by the Dr. van Haeringen laboratorium B.V., Wageningen, NL. A linkage map was constructed using the JoinMap 4.1 software (Van Ooijen 2006). Map distances were calculated using the Kosambi mapping function.

### QTL mapping

Potential resistance QTLs associated with aphid survival and reproduction were identified using the MapQTL 6.0 software (Van Ooijen 2011). Interval mapping analysis was first performed to look for regions with potential QTL effects. Multiple-QTL mapping (MQM) was applied to find additional QTLs using the top marker in the major QTL region as co-factor. A permutation test was used to determine the LOD threshold for aphid survival and reproduction corresponding to a genome-wide confidence level of 0.05. The QTL graphs were drawn using MapChart 2.3, including a 2-LOD confidence interval (Voorrips 2002).

### Fine mapping

Leaf samples of 1118 plants from one F_3_ line (2,016,027) were genotyped using the KASP™ technology (KBioscience, UK) with 21 SNP markers in the 2-LOD confidence interval of the major QTL. Seven additional SNP markers were designed and used for further genotyping through the LightScanner System (Idaho Technology, USA) (Wittwer et al. 2003). Primers were designed using Primer3 (Untergasser et al. 2007). The PCR products that were used for the LightScanner were amplified according to the protocol of the manufacturer. All used markers are listed in Table S1.

## Results

### Aphid performance on the F_2_ population and parents

**Fig. 1:**
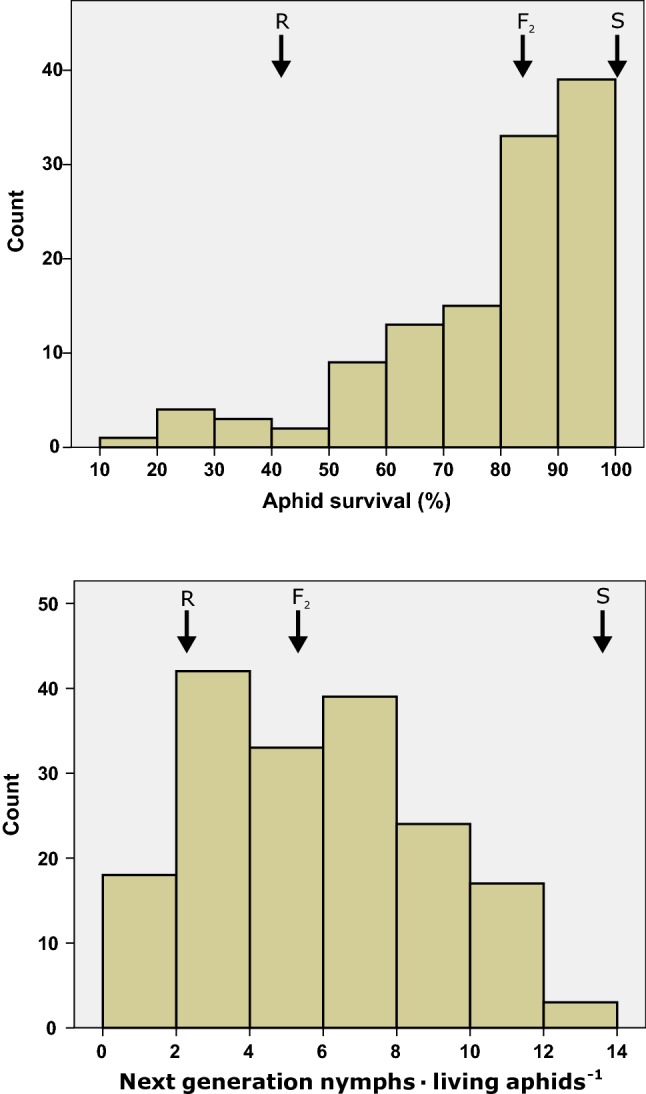
Aphid resistance in the F_2_ population. Frequency distribution of survival of original aphids (top) and the aphid reproduction (bottom) in the 176 F_2_ plants for which genotyping and phenotyping data were obtained. The population was derived from a cross between an aphid-resistant and susceptible *Capsicum baccatum* plant. Black arrows indicate the approximate means of the resistant (*R*) and susceptible (*S*) parental inbred line and the F_2_ population

We monitored GPA performance on the F_2_ population and the first-generation parental inbred lines (resistant line PB2014009 and susceptible line PB2014005). The two inbred lines showed significant differences in aphid performance for the two parameters used: survival of the original nymphs (42 ± 13% vs. 97 ± 4%; *T* test, *P* < 0.001) and number of next-generation nymphs produced per aphid (2.2 ± 0.8 vs. 13.8 ± 1.7; *T* test, *P* < 0.001), on resistant and susceptible line, respectively. The F_2_ population showed a large variation for GPA performance based on the two parameter used: 20–100% survival of the aphids placed on the plant and 0.2–13.3 new nymphs produced per aphid (Fig. 1). The correlation between the two used parameters was *R* = 0.72 (Pearson correlation, *P* < 0.001). The average aphid survival (84%) and the average number of new nymphs per aphid (5.7) in the F_2_ population were somewhat skewed towards the susceptible and resistant inbred line, respectively.

### Linkage map

A genetic linkage map was constructed with 176 F_2_ individuals (16 out of 192 plants were discarded because of incomplete data) and 167 SNP markers (Figure S1, Table S2). The constructed 12 linkage groups, which correspond to the number of pepper chromosomes, varied in length from 85 cM (LG2) to 139 cM (LG11), with a total length of 1319 cM and an average distance of 8 cM between markers. The largest gap between two markers was 33 cM and located on LG8. The assignment of linkage groups was according to the chromosomal location of the SNP markers that were most frequently found in a group and also according to the BLAST result of SNP markers to the genome of *C. baccatum* ‘PBC81’ (Kim et al. 2017). Linkage group 3 of *C. baccatum* contained segments of chromosome 3 and 9 of *C. annuum*, LG5 of chromosomes 3 and 5 and LG9 contained segments of chromosome 3, 5 and 9 of *C. annuum*. The other linkage groups seemed to be homologous to the chromosomes of *C. annuum* (Figure S1).

### QTL mapping

**Fig. 2:**
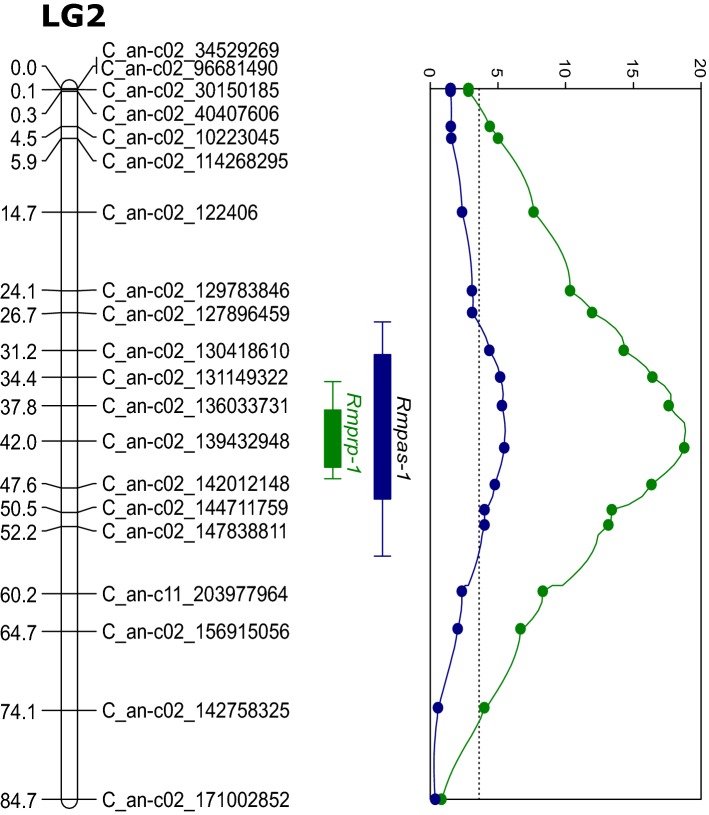
Linkage map, LOD profiles and 1-LOD and 2-LOD support intervals for aphid resistance QTLs on linkage group 2. Blue and green lines represent the profiles for survival of original aphids and aphid reproduction, respectively. The dotted line at LOD 3.5 represents the LOD threshold. LG2, linkage group 2, is assigned to chromosome 2 of the pepper genome. *Rmpas-1* and *Rmprp-1* represent major resistance QTL for aphid survival and reproduction, respectively. The *y*-axis of the LOD profile shows the LOD score. The number at the end of the marker name indicates its physical position on the CM334 v.1.55 genome (Kim et al. 2014) (colour figure online)

Interval mapping of aphid survival and reproduction resulted in the identification of QTLs for both of them, which we designated *Rmpas-1* and *Rmprp-1*, respectively. Both QTLs were located on chromosome 2 (LG2, Fig. 2), with marker C_an-c02_139432948 as top marker. The LOD scores at this marker were 5.5 and 18.8, with an explained phenotypic variance of 14.7% and 41.9% for survival and reproduction, respectively (Table 1). The 2-LOD intervals span 25.5 cM (between markers C_an-c02_127896459 and C_an-c02_147838811) for survival and 13.2 cM (between markers C_an-c02_131149322 and C_an-c02_142012148) for reproduction. One additional, minor QTL for reproduction was detected on chromosome 4 (LG4, Figure S2) using Multiple QTL Model (MQM) mapping with marker C_an-c02_139432948 as co-factor. This minor QTL (*Rmprp-2*) explained 6.4% of phenotypic variation (Table 1). No epistasis was detected between the major QTL *Rmprp-1* and the minor QTL *Rmprp-2*.

**Table 1:** QTL effect of resistance-related traits after the infestation with *M. persicae* determined in the F_2_ population

Traits	QTL name	Marker at QTL peak	Chromosome	Position (cM)^a^	LOD	Additive effect^b^	Dominance effect^c^	% Explained variance
Aphid survival^d^	*Rmpas-1*	C_an-c02_139432948	2	42.0	5.47	0.16	0.32	14.7
Aphid reproduction^−1e^	*Rmprp-1*	C_an-c02_139432948	2	42.0	18.76	0.66	− 0.09	41.9
	*Rmprp-2*	C_an-04_30341348	4	46.3	4.06	0.26	0.25	6.4

^a^Genetic position of the QTL in the linkage group

^b^Positive values indicate that alleles from susceptible accession result in higher phenotypic values than those from resistant accession

^c^Positive values indicate that the heterozygote condition results in higher phenotypic values than the midparent value

^d^Based on arcsin(sqrt(*x*)) transformed data

^e^Based on sqrt(*x*) transformed data

### Confirmation of the resistance QTLs on chromosome 2 in F_3_ lines

**Fig. 3:**
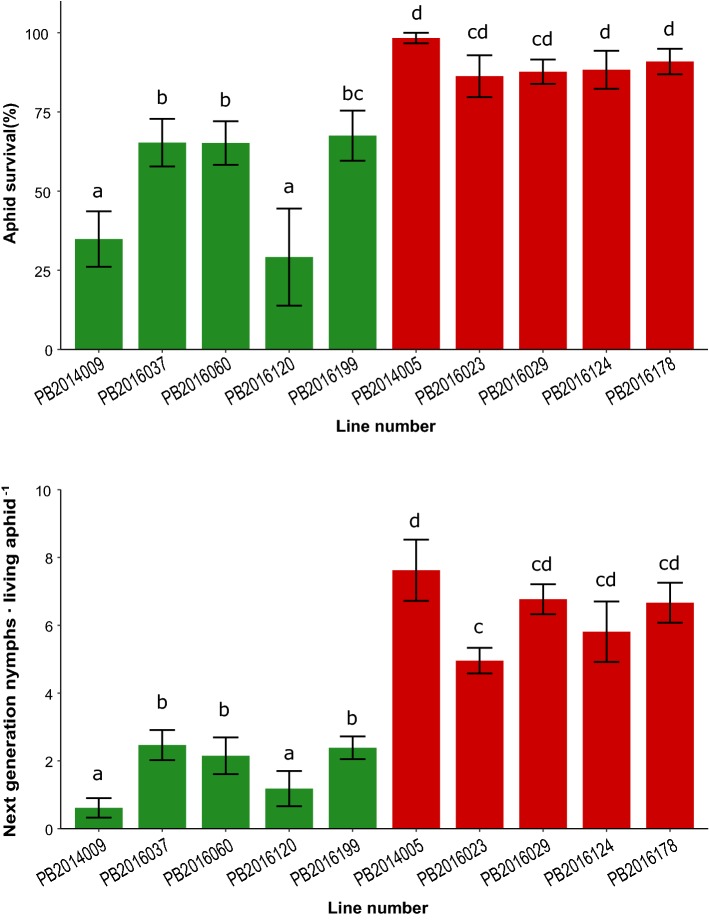
Performance of *M. persicae* on F_3_ lines and two parental inbreds. Four F_3_ lines (PB2016037, PB2016060, PB2016120 and PB2016199) originating from four F_2_ plants homozygous for the resistance allele and four F_3_ lines (PB2016023, PB2016029, PB2016124 and PB2016178) homozygous for the susceptibility allele in the 2-LOD interval of the major QTL *Rmpas-1* and *Rmprp-1* on chromosome 2. The inbred lines PB2014009 and PB2014005 were obtained by self-pollination of the resistant and susceptible parent of the F_2_ population, respectively. Each bar represents the mean values ± standard error. Green and red bars represent plants with genotypic background homozygous for resistance and susceptibility allele, respectively. Same letters indicate that values are not significantly different (LSD test on transformed scales at *P* < 0.05) (colour figure online)

The effect of the major QTL on both aphid survival and reproduction was validated in a set of eight F_3_ lines, originating from four F_2_ plants homozygous for the resistance allele (lines 2,016,037, 2,016,060, 2,016,120 and 2,016,119) and four plants homozygous for the susceptibility allele (lines 2,016,023, 2,016,029, 2,016,124 and 2,016,178) in the 2-LOD interval around the top marker on chromosome 2 (Fig. 2). Aphids feeding on all lines with the resistance allele in the QTL region produced significantly fewer new nymphs than aphids feeding on lines without the resistance allele on chromosome 2 (Fig. 3). For aphid survival, the difference between lines with the resistance allele and lines with the susceptibility allele was not as clear as the difference in aphid reproduction. There was no significant difference in aphid survival on line PB2016199 which has the resistance allele in the QTL region and the lines PB2016023 and PB2016029 which have the susceptibility allele.

### Fine mapping of QTL *Rmprp-1*

To fine map the major QTL *Rmprp-1* affecting aphid reproduction, we genotyped 1118 plants from F_3_ line PB2016027 with marker C_an-c02_131149322 and marker C_an-c02_142012148 flanking the 2-LOD interval, together with 19 extra markers (Table S1) to identify recombinants. Five plants that were homozygous for the resistance or susceptibility allele of *Rmprp-1* and five plants of both parental inbred lines were phenotyped together with the 230 recombinants. The two sets of homozygous F_3_ plants showed a significantly different reproduction: 3.2 ± 0.9 versus 7.8 ± 0.6 new nymphs per aphid (*T* test, *P* < 0.001). There was no significant difference in reproduction between homozygous susceptible plants from F_3_ line PB2016027 and plants from inbred line PB2014005 [7.8 ± 0.6 vs. 8.6 ± 1.4 (*T* test, *P* = 0.254)]. Plants from inbred line PB2014009 were more resistant than homozygous resistant plants from F_3_ line PB2016027 [0.9 ± 0.9 vs. 3.2 ± 0.9 (*T* test, *P* = 0.003)].

**Fig. 4:**
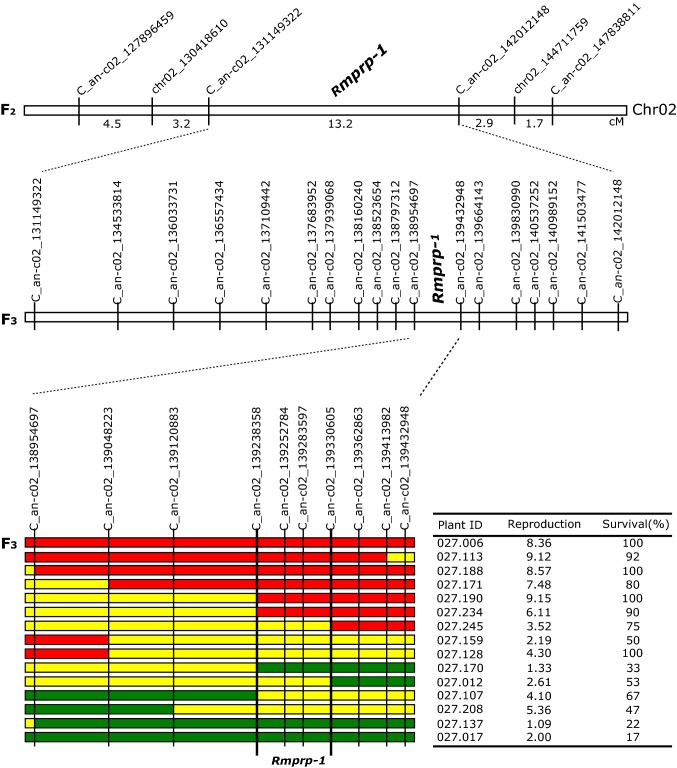
Fine mapping of the major resistance QTL *Rmprp-1*. The genotype in the area between marker 138,954,697 and marker Chr02_139432948 of F_3_ plants is shown in colour codes (red = homozygous susceptible allele, yellow = heterozygous, and green = homozygous resistant allele). The number at the end of the marker name indicates its physical positions on the CM334 v.1.55 genome (Kim et al. 2014). The three columns behind the coloured bars indicate ID number of the F_3_ plant, average number of next-generation nymphs produced per aphid and survival of the original aphids (%) (colour figure online)

Among 1118 plants from F_3_ line PB2016027, 230 plants had a recombination between the 2-LOD flanking markers. The estimated distance between C_an-c02_131149322 and C_an-c02_142012148 was 11.9 cM (in the F_2_ population: 13.2 cM), and the relative order of the markers and genetic distances between them was consistent with their position on the physical map of chromosome 2 of *C. annuum* ‘CM334’ and also with their position on chromosome 2 of *C. baccatum* ‘PBC81’ (Kim et al. 2017) based on BLAST results. After phenotyping all the 230 recombinants, aphid resistance QTL *Rmprp-1* was mapped to an area between marker C_an-c02_138954697 and marker C_an-c02_139432948 (Fig. 4). As there were still 15 recombinants in this area, seven additional SNPs markers were developed to genotype these 15 recombinants. This resulted in a further fine mapping of *Rmprp-1* to a 92.3 kb area between marker C_an-c02_139238358 and marker C_an-c02_139330605 (Fig. 4). The physical distance between these two markers is 92.3 kb in the genome of *C. annuum* (Kim et al. 2014) and 96.2 kb in the genome of *C. baccatum* ‘PBC81’. In the genome of ‘PBC81’ (www.ncbi.nlm.nih.gov/nuccore/CM008444.1?report=graph), six genes are identified in the target region and four of them are annotated as ‘probable LRR receptor-like serine/threonine-protein kinase’ (Table 2).

**Table 2:** Annotated genes in the area of major QTL *Rmprp-1* in pepper genome ‘PBC81’

Gene ID	Location (bp)^a^	CDS length (bp)^b^	Functional annotation
*CQW23_04318*	129,922,179–129,941,715	3036	LRR receptor-like serine/threonine-protein kinase
*CQW23_04319*	129,951,251–129,954,463	810	unknown
*CQW23_04320*	129,971,136–129,975,767	552	LRR receptor-like serine/threonine-protein kinase
*CQW23_04321*	129,981,447–129,990,787	2076	LRR receptor-like serine/threonine-protein kinase
*CQW23_04322*	129,991,551–130,002,579	3003	LRR receptor-like serine/threonine-protein kinase
*CQW23_04323*	130,011,293–130,014,738	663	unknown

^a^Physical position of the gene on chromosome 2 based on the assembled genome from NCBI

^b^CDS indicates coding sequence

## Discussion

### Aphid resistance QTLs

A SNP-based linkage map of *C. baccatum* was constructed for QTL mapping of aphid resistance. The map contains 12 linkage groups, which is identical to the number of pepper chromosomes and covers a total length of 1319 cM, which is similar to some other published maps of either *C. annuum* or *C. baccatum* (Eun et al. 2016; Lee et al. 2016; Mahasuk et al. 2016). Comparing our linkage map to the reference physical genome of *C. annuum* ‘CM334’(Kim et al. 2014) pointed at translocations between chromosomes 3 and 5, and chromosomes 3 and 9, which is consistent with previous observations (Lee et al. 2016; Mahasuk et al. 2016). However, we did not find the translocation between chromosomes 1 and 8, which was previously detected (Lee et al. 2016). The reason for this might be the small number of markers on these two chromosomes in our study. Although the markers used were evenly distributed based on their physical position on the reference genome, there were still a number of gaps in the genetic map, some of which were around 30 cM. It is possible that minor QTLs located in these gaps have not been detected.

This is the first time that an aphid resistance QTL has been mapped in pepper. Two parameters were used to quantify resistance: survival of original aphids put on the plant and number of new nymphs produced by each of these aphids. The two parameters were significantly correlated (Pearson correlation, *R* = 0.72, *P* < 0.001). Therefore, it is not surprising that the major QTLs, *Rmpas-1* and *Rmprp-1*, share the same region on chromosome 2.

The major QTL *Rmprp-1* explained about 42% of the variance for the production of next-generation nymphs. In addition, a minor QTL *Rmprp-2* was detected on chromosome 4, which explains approx. 6% of the variance. The QTL *Rmprp-1* has been validated using F_3_ lines as reported here and also using F_4_ and F_5_ lines (data not shown). However, the minor QTL *Rmprp-2* still needs validation. As the dominance effect of the major QTL *Rmprp-1* is negative (− 0.09; Table 1) and much smaller than its additive effect (0.66), the QTL for aphid reproduction can be regarded as partially dominant for resistance. The QTL for aphid survival (*Rmpas-1*) was also located on chromosome 2 and explains about 15% of the variance in the F_2_; it was also validated using F_3_ lines. No other QTLs for aphid survival were detected. The unexplained F_2_ variance might be due to environmental variation and/or to several undetected small effects of QTLs segregating in this F_2_ population. Segregation of undetected small effects QTLs may cause some F_3_ plants to deviate in their phenotype from the expected values based on the major QTL. This is for instance supported by the observation that a similar GPA survival was found with one F_3_ line that was homozygous resistant (line PB2016199) and two F_3_ lines which were homozygous susceptible (line PB2016023 and PB2016029) for the QTL on chromosome 2. Although the major QTL explained only 15% of the F_2_ variance for aphid survival, in the QTL validation experiment the difference between the F_3_ lines with the resistance and the susceptibility alleles was about 50% of the difference between the parental inbred lines. This suggests that the low percentage of explained F_2_ variance for aphid survival may be due to environmental variation. Environmental variation may affect aphid survival more than reproduction. One reason could be that adaptation of the nymphs to their new environment (clip cage, new genotype) may affect survival, while reproduction usually takes place about 8 days after start of the clip cage test, when aphids are already adapted to their new ‘home’. A second reason could be that handling of the small nymphs may cause varying amounts of damage/stress leading to increased aphid mortality. As the reproduction is calculated based on the average number of living aphids, the effects of handling on mortality are partially compensated leading to a lower environmental variation and a higher fraction of explained variance. This QTL, with a large effect on reproduction and a smaller effect on adult survival, is the first QTL described in pepper which is related with antibiosis resistance. Antibiosis-based resistance is helpful in reducing the build-up of an aphid population (Züst and Agrawal 2016). Preventing aphid population build-up may also interfere with the spread of some viruses (Radcliffe and Ragsdale 2002).

As the detected QTLs are located on chromosomes 2 and 4, where no translocations have been found between *C. baccatum* and *C. annuum*, it looks feasible to transfer the resistance QTLs from *C. baccatum* to commercial *C. annuum* cultivars by hybridization. However, because of the post-fertilization barriers between *C. baccatum* and *C. annuum* (Eshbaugh 1970), the necessary interspecific crosses are likely to need some effort (Yoon et al. 2005).

### Resistance QTL *Rmprp-1* is mapped to a cluster of receptor-like kinase genes

The major resistance QTL *Rmprp-1* was fine mapped to a 96 kb region based on the recently released *C. baccatum* ‘PBC81’ genome located between markers C_an-c02_139238358 and C_an-c02_139330605 on chromosome 2. During fine mapping of QTL *Rmprp-1*, the 230 plants from F_3_ line 2,016,027 were not only phenotyped for reproduction but also for survival. The two traits showed a similar correlation as in the F_2_ (*R* = 0.73, Pearson correlation *P* < 0.001). It seems likely that QTL *Rmpas-1* and *Rmprp-1* are based on the same causal gene, although it cannot be excluded that they are due to two different but very closely linked genes. The current annotation of the 96 kb region predicts the presence of six putative genes. Two genes are annotated as ‘unknown’, the other four as receptor-like kinase genes with a leucine-rich repeat domain (LRR-RLKs), suggesting that the gene underlying the resistance QTL for aphid reproduction (and probably aphid survival) may belong to the LRR-RLK family.

The LRR-RLK family is the major group of plant receptor like kinases (Shiu and Bleecker 2001). This type of genes encodes a protein containing a receptor domain for signal perception and a single-pass transmembrane domain for protein anchoring, together with a cytoplasmic serine/threonine-protein kinase domain for signal transduction (Shiu and Bleecker 2001). One of the most important functions of identified plant LRR-RLKs is responding to environmental stress and subsequent induction of plant defences (Sakamoto et al. 2012; Shiu and Bleecker 2001). These LRR-RLKs can recognize microbe-associated molecular patterns (MAMPs) and are required for MAMP-triggered immunity (Silva Couto and Zipfel 2016). Among the LRR-RLKs in the Solanaceous crops, the LRR-RLK FLS2 is found to perceive MAMP flg22 from bacteria and activate an immunity response in tomato (Robatzek et al. 2007). Two paralogs SlSERK3A and SlSERK3B in tomato have distinct but also overlapping functions in bacterial and nematode innate immunity (Peng and Kaloshian 2014). To our best knowledge, no solid evidence is available at present that any LRR-RLK gene recognizes herbivore-associated molecular patterns (HAMPs) from aphids and contributes to aphid resistance, although a cucumber LRR-RLK was found to be the most likely candidate gene in the defence response to *Aphis gossypii* (Liang et al. 2016).

As four annotated *LRR-RLK* genes are located in the 96 kb area on chromosome 2 and sequence similarity among the genes is high, we can consider them as a gene cluster (Graham 1995). The *LRR-RLK* genes often occur in clusters consisting of several homologous genes (Shiu and Bleecker 2001; Wei et al. 2015; Zhou et al. 2016). Clustering of *LRR-RLK* genes, which is probably caused by gene duplications, was suggested to be a consequence of adaptation to fast-evolving biotic stresses (Lehti-Shiu et al. 2009). Multiple genes in a cluster may allow fast selection for the detection of diverse biotic attackers. However, the high similarity among these genes and their close proximity make it difficult to identify the one that is conferring the resistance to GPA. Further efforts are needed to identify and validate the gene conferring aphid resistance and to elucidate the resistance mechanism.

## Conclusion

In this study, we have mapped for the first time QTLs conferring resistance to GPA in pepper. The QTL region was narrowed down to a gene cluster with four analogues of the LRR-RLK subfamily. This work will significantly speed up the breeding of aphid-resistant pepper varieties.

## Electronic supplementary material

Below is the link to the electronic supplementary material.

Figure S1. Genetic linkage map of Capsicum baccatum. The map is based on 167 SNP markers segregating in an F2 population of 192 plants, which was derived from a cross between an aphid resistant and susceptible C. baccatum plant. The 12 linkage groups LG1–LG12 correspond to chromosomes 1–12 of C. baccatum (Kim et al. 2017) (PDF 26 kb)

Figure S2. Linkage map, LOD profiles and 1-LOD and 2-LOD support intervals for the minor aphid resistance QTL on linkage group 4. Blue and green lines represent the profiles for survival of the aphids that were placed on the plant, and number of new nymphs produced per aphid, respectively. The dotted line at LOD 3.5 represents the LOD threshold. LG4, linkage group 4, is assigned to chromosome 4 of the pepper genome. Rmprp-2 represents a minor resistance QTL for aphid reproduction. The y-axis of the LOD profile shows the LOD score (PDF 26 kb)

Supplementary file 3 (XLSX 29 kb)

Supplementary file 4 (DOCX 15 kb)
