# Supplementary figures and images for: Aphid resistance in Capsicum maps to a locus containing LRR-RLK gene analogues
Source: Theor Appl Genet. 2019 Oct 8;133(1):227–37. doi: 10.1007/s00122-019-03453-7 (PMC6952328; doi:10.1007/s00122-019-03453-7)

LG1

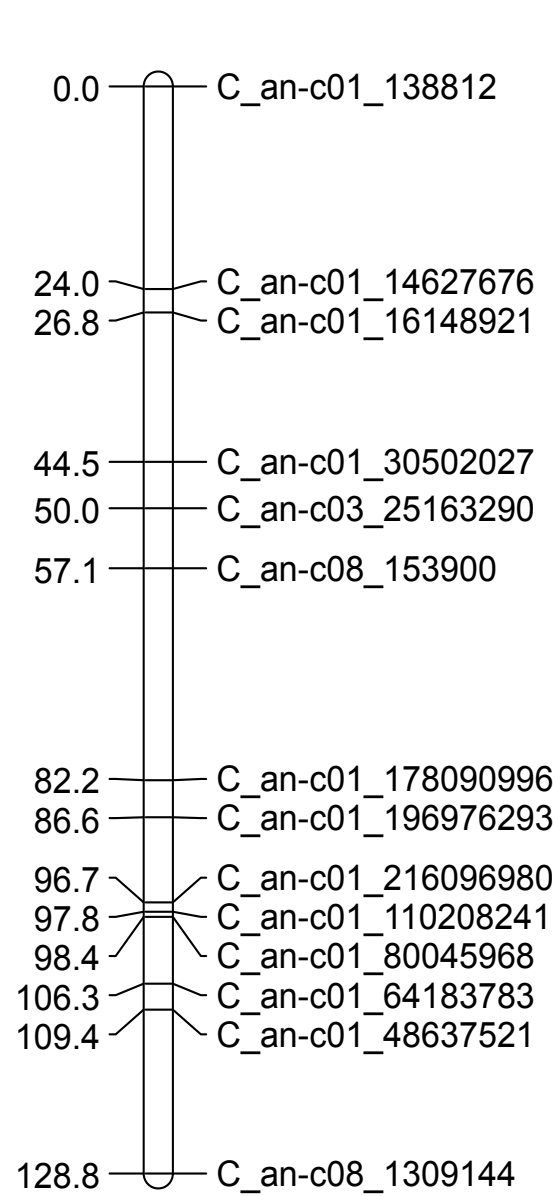

LG2

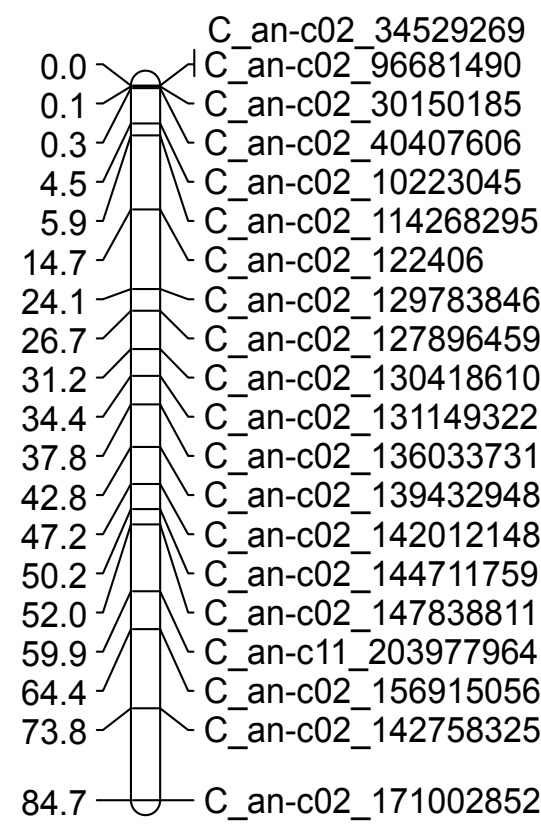

LG3

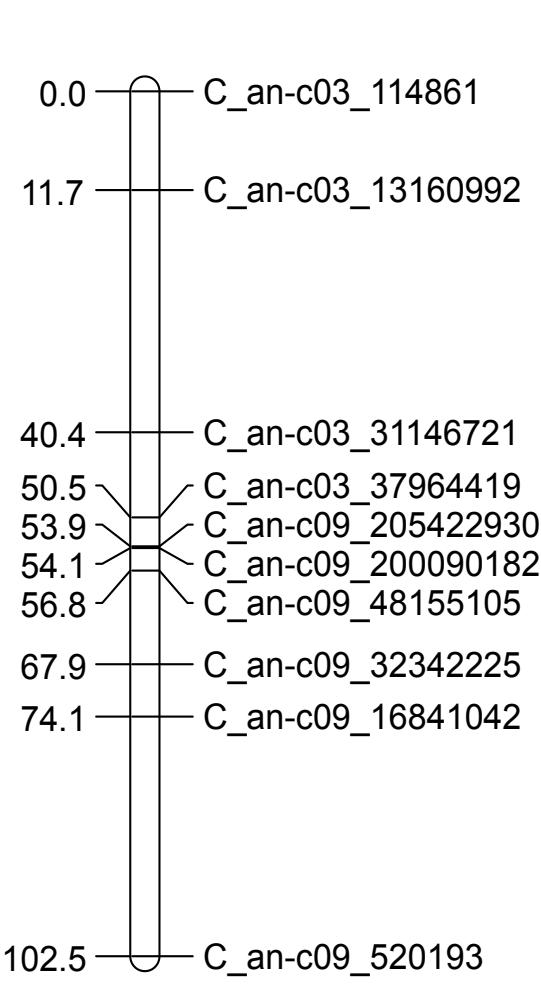

LG4

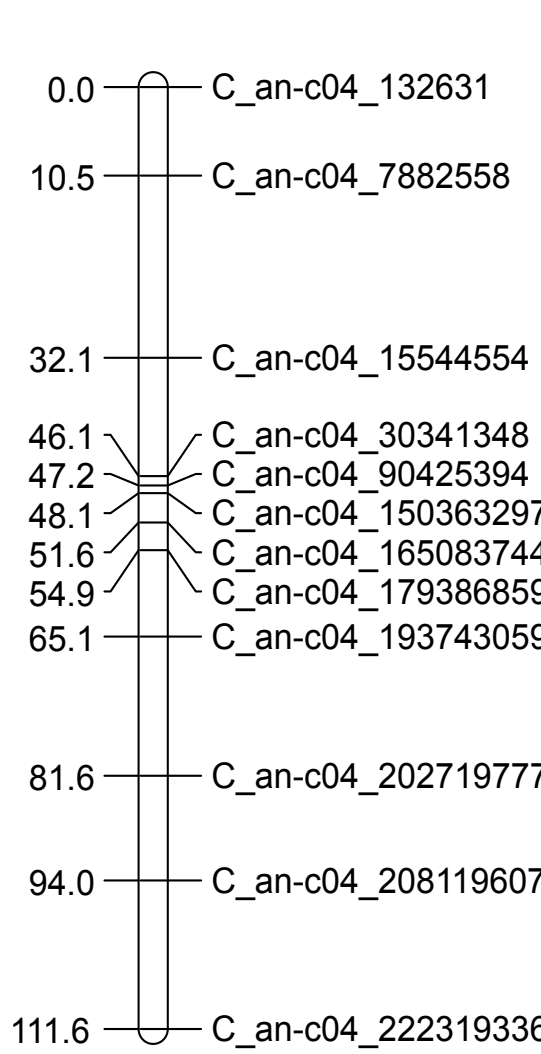

LG5

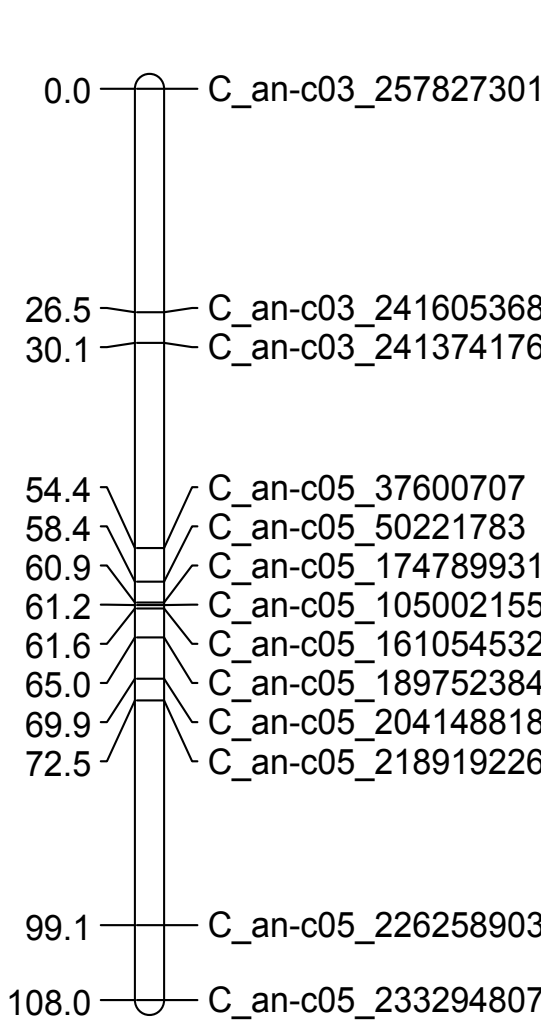

LG6

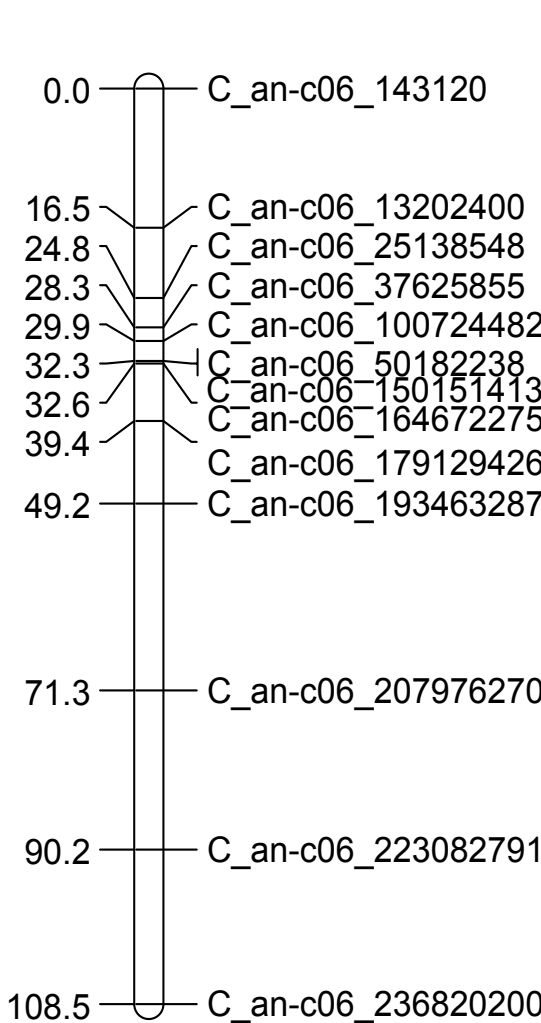

LG7

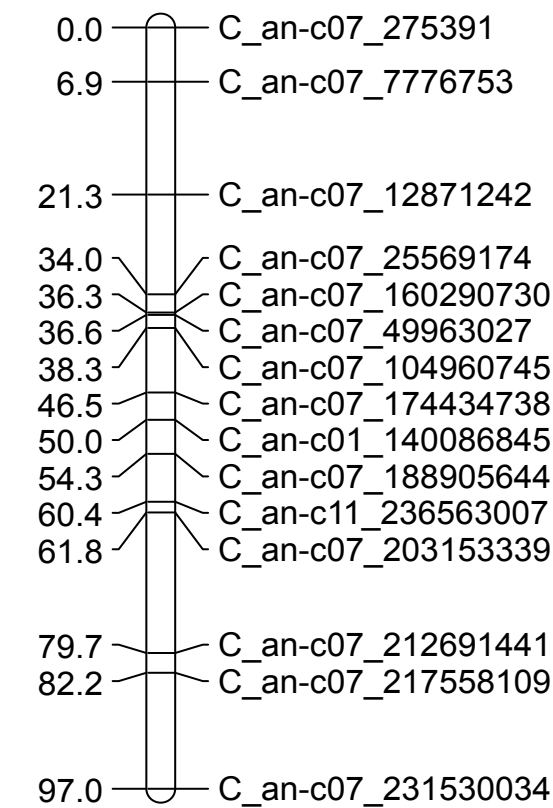

LG8

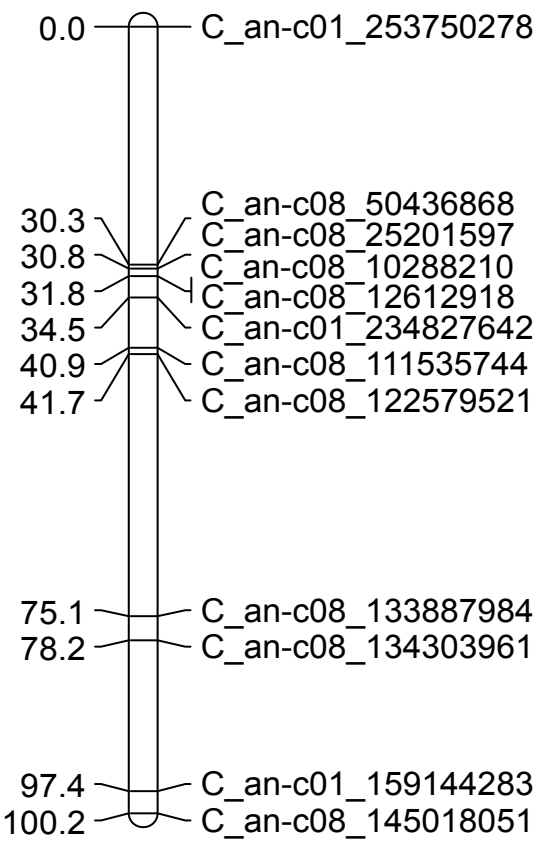

LG9

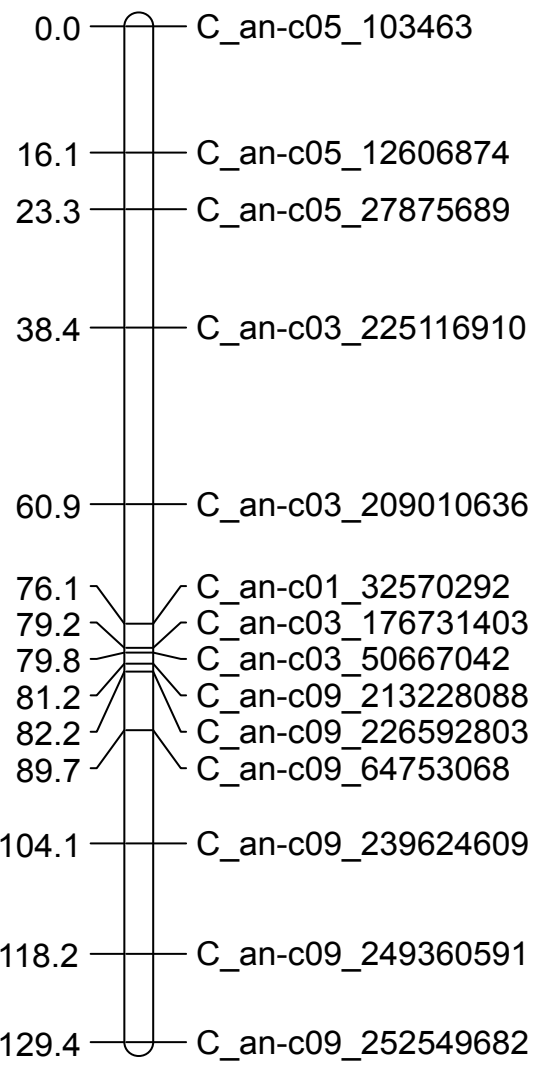

LG10

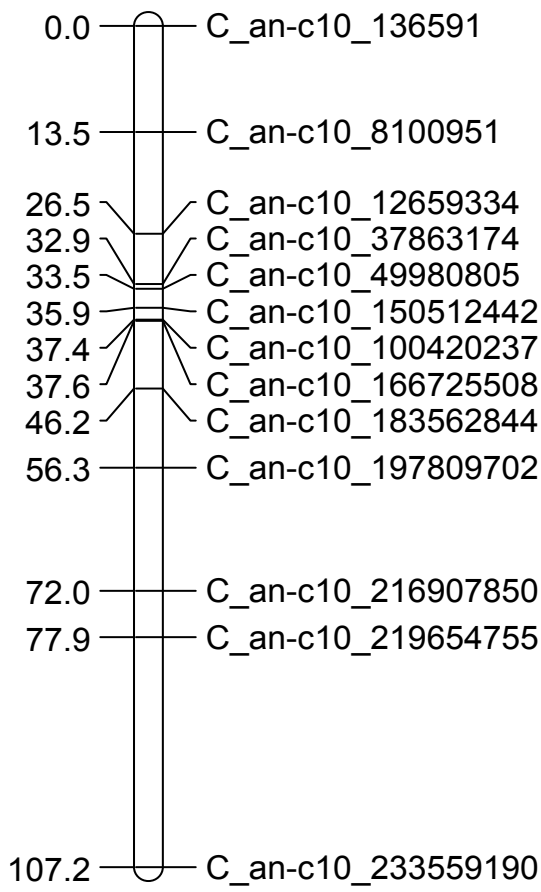

LG11

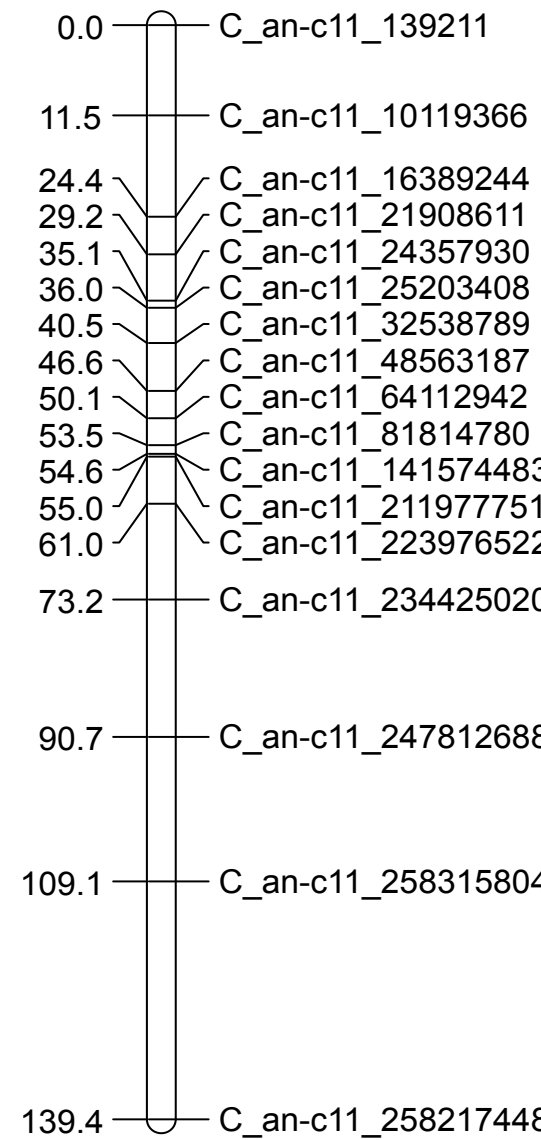

LG12

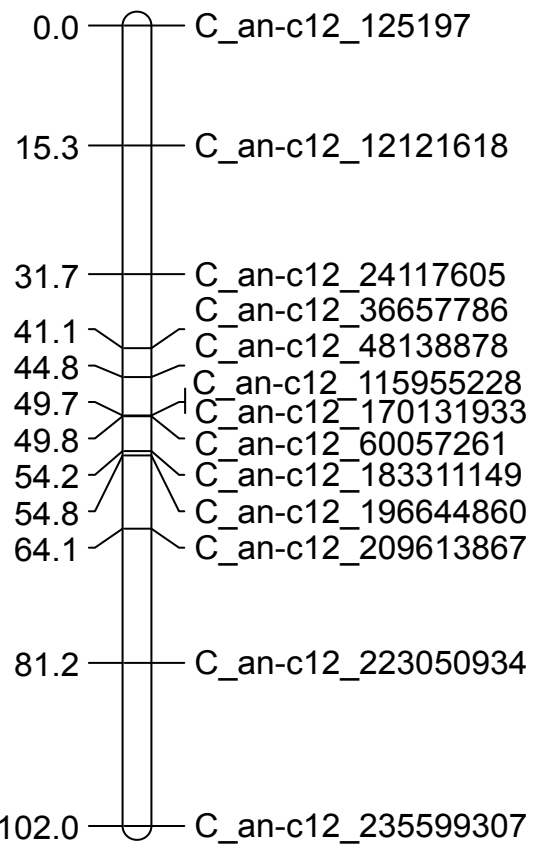

Supplement: Supplementary file 1 — Figure S1. Genetic linkage map of Capsicum baccatum. The map is based on 167 SNP markers segregating in an F2 population of 192 plants, which was derived from a cross between an aphid resistant and susceptible C. baccatum plant. The 12 linkage groups LG1–LG12 correspond to chromosomes 1–12 of C. baccatum (Kim et al. 2017) (PDF 26 kb) [file 122_2019_3453_MOESM1_ESM.pdf]

# LG4

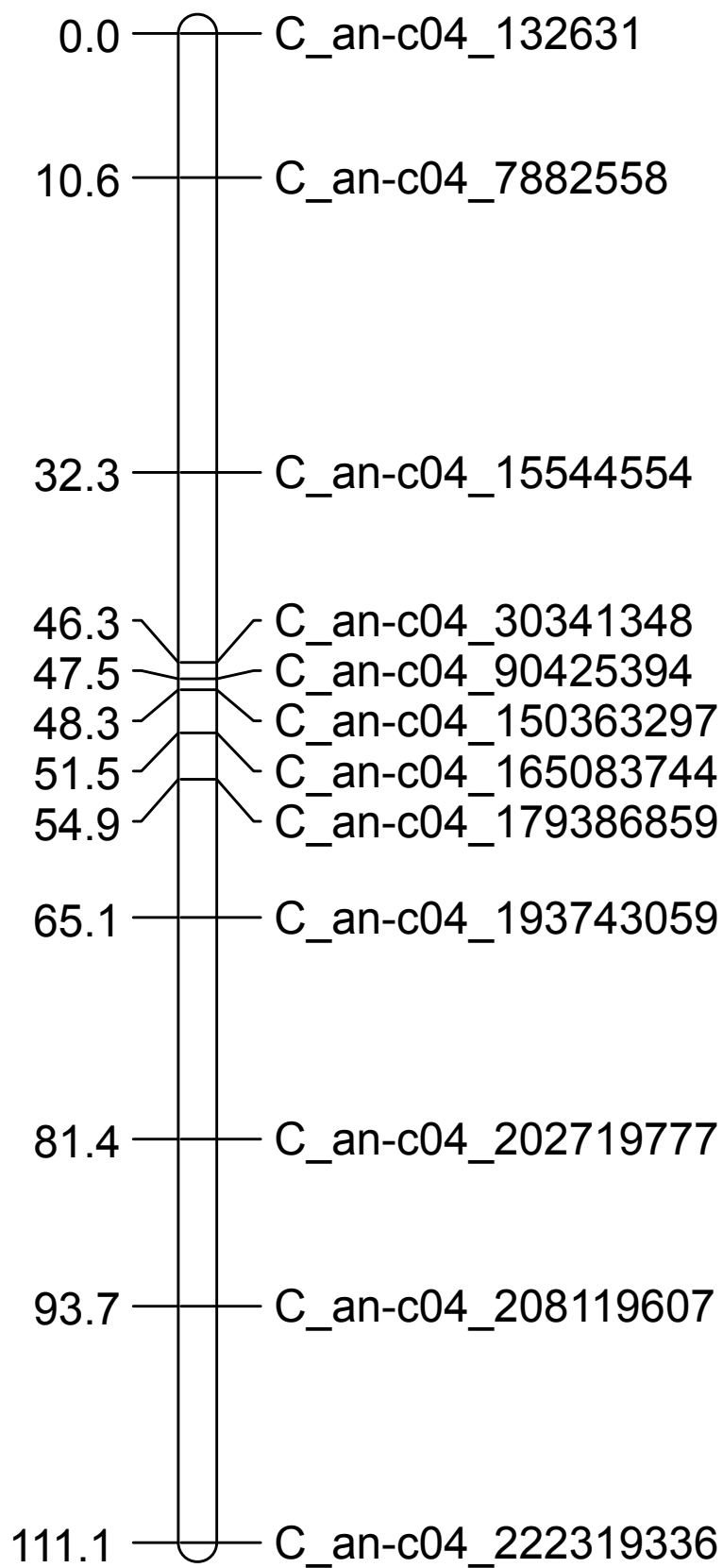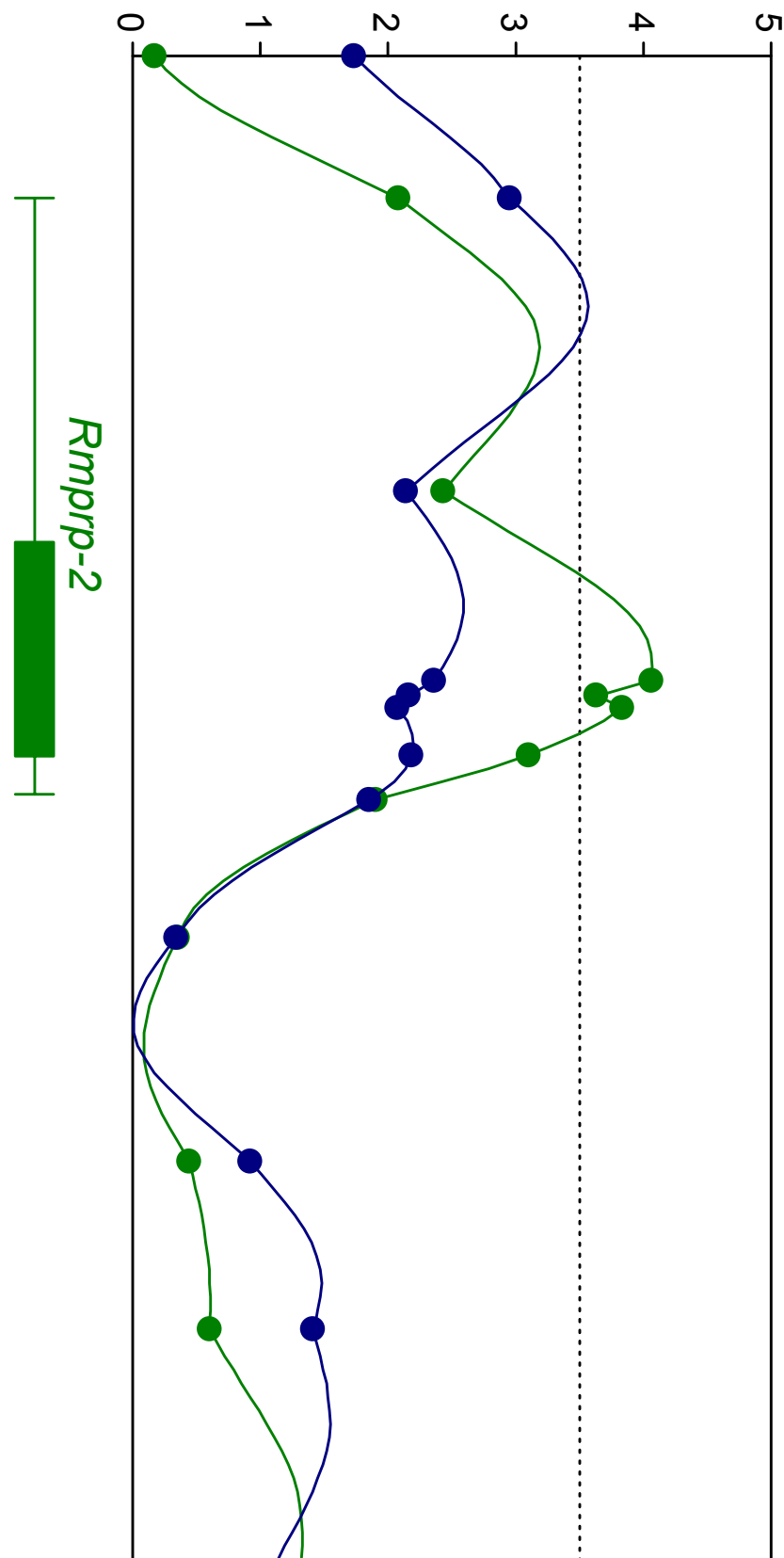

Supplement: Supplementary file 2 — Figure S2. Linkage map, LOD profiles and 1-LOD and 2-LOD support intervals for the minor aphid resistance QTL on linkage group 4. Blue and green lines represent the profiles for survival of the aphids that were placed on the plant, and number of new nymphs produced per aphid, respectively. The dotted line at LOD 3.5 represents the LOD threshold. LG4, linkage group 4, is assigned to chromosome 4 of the pepper genome. Rmprp-2 represents a minor resistance QTL for aphid reproduction. The y-axis of the LOD profile shows the LOD score (PDF 26 kb) [file 122_2019_3453_MOESM2_ESM.pdf]
