# Supplementary material for: Aphid resistance in Capsicum maps to a locus containing LRR-RLK gene analogues
Source: Theor Appl Genet. 2019 Oct 8;133(1):227–37. doi: 10.1007/s00122-019-03453-7 (PMC6952328; doi:10.1007/s00122-019-03453-7)
Supplement: Supplementary file 4 — (DOCX 15 kb) [file 122_2019_3453_MOESM4_ESM.docx]

**Table S2. Summary of the genetic linkage map of *C. baccatum.*** The map was constructed from an F_2_ population originated from a cross between an aphid resistant and susceptible plant. Also indicated is the corresponding *C. annuum* chromosome (Kim et al, 2014).

| **Linkage group** | **Number of SNP markers** | **Length of the linkage group (cM)** | **Corresponding chromosome(s)**  **of *C. annuum*** |
| --- | --- | --- | --- |
| LG1 | 14 | 129 | Chr.1 |
| LG2 | 20 | 85 | Chr.2 |
| LG3 | 10 | 103 | Chr.3, Chr.9 |
| LG4 | 12 | 112 | Chr.4 |
| LG5 | 13 | 108 | Chr.3, Chr.5 |
| LG6 | 13 | 109 | Chr.6 |
| LG7 | 15 | 97 | Chr.7 |
| LG8 | 13 | 100 | Chr.8 |
| LG9 | 14 | 129 | Chr.3, Chr.5, Chr.9 |
| LG10 | 13 | 107 | Chr.10 |
| LG11 | 17 | 139 | Chr.11 |
| LG12 | 13 | 102 | Chr.12 |
| Total | 167 | 1319 | - |
